# Conditional survival of metastatic clear cell renal cell carcinoma: How prognosis evolves after cytoreductive surgery of primary tumor

**DOI:** 10.1002/cam4.4270

**Published:** 2021-09-12

**Authors:** Haixiang Shen, Jin Liu, Wei Liu, Jiazhu Sun, Xiangyi Zheng, Lisong Teng, Xiao Wang, Liping Xie

**Affiliations:** ^1^ Department of Urology First Affiliated Hospital Zhejiang University School of Medicine Hangzhou China; ^2^ Department of Surgical Oncology First Affiliated Hospital Zhejiang University School of Medicine Hangzhou China; ^3^ Department of Urology Zhongnan Hospital of Wuhan University Wuhan China

**Keywords:** clear cell, conditional survival, cytoreductive surgery, metastatic renal cell carcinoma

## Abstract

**Introduction:**

Cytoreductive surgery is one of the recommended treatments for metastatic renal cell carcinoma, while the prognostic information of these patients treated with cytoreductive surgery is limited. In this study, we aimed to investigate the survival profiles based on conditional survival (CS) estimates in metastatic clear cell renal cell carcinoma (mccRCC) patients treated with cytoreductive surgery of primary tumor.

**Methods and materials:**

We identified and extracted mccRCC patients from the Surveillance, Epidemiology, and End Results database. We used Kaplan–Meier method to perform CS analyses. A multivariate Cox regression model was applied to explore the changes of well‐known prognostic factors.

**Results:**

Conditional overall survival (COS) and conditional cancer‐specific survival (CCSS) improved increasingly at all periods of survivorships compared to survival estimates at baseline in overall population of mccRCC. The 36‐month COS improved by 3.3%–6.4% given per 12 additional months of survivorships and the CCSS improved significantly from 45.1% (95% CI 42.8–47.3) at 12 months to 67.1% (95% CI 62.0–71.7) at 60 months. Much more survival gain was observed in patients with advanced disease. Furthermore, the prognostic significance of age and pathological factors diminished and even disappeared in a long‐term survivorship.

**Conclusions:**

Conditional overall survival and CCSS improved with time dynamically in mccRCC patients treated with cytoreductive surgery of primary tumor. Patients with advanced disease achieved significant survival gain and even could harvest a better prognosis given that the time of survivorship exceeds a certain period. Our findings could provide valuable and practical data for patient counseling and surveillance strategy making.

## Graphical abstract

In this study, we investigated the survival profiles based on conditional survival estimates in metastatic clear cell renal cell carcinoma (mccRCC) patients treated with cytoreductive surgery of primary tumor. We found that COS and CCSS improved with time dynamically in all mccRCC patients treated with cytoreductive surgery of primary tumor.

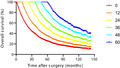

## 1 INTRODUCTION

Renal cell carcinoma (RCC) is one of the common cancers in the urinary system with a major histological type of clear cell renal cell carcinoma (ccRCC) (~80%).^1^ About 403,262 (2.2%) newly diagnosed RCC patients and 175,098 (1.8%) mortalities were estimated in 2018.^2^ Although more and more small renal masses are detected at an early stage by routine use of imaging modalities, 15%–30% of patients present with metastatic RCC (mRCC) at initial diagnosis.^3^, ^4^ And ~30% of localized RCC eventually progress to metastasis suffering poor prognosis.^5^ Five‐year disease‐specific survival for mRCC patients with low‐ and intermediate‐risk are only 32% and 19.5%, respectively.^6^

Generally, estimated survival outcomes at initial diagnosis like 5‐year survival rate are employed to counsel patients on their prognosis and make follow‐up schemes. However, the survival probability changes dynamically over time and this traditional prediction approach only presents an estimated survival probability at a certain time without considering changeable value of risk factors as time advances. As reported in some studies, crucial prognostic factors identified at diagnosis might fail to affect the prognosis of patients with malignancies as the time of survivorship increases.^3^, ^7^ Therefore, conditional survival (CS) is introduced to provide more precise and personalized prediction of prognosis in consideration of the duration patients have already survived.

The application of CS estimates models was reported in various malignancies like pancreatic adenocarcinoma,^8^ colorectal carcinoma,^9^, ^10^ prostate cancer,^11^ bladder cancer^7^ and malignant hematologic diseases.^12^ As for CS on mRCC, previous studies mainly focused on metastatic non‐ccRCC or patients treated with targeted therapy.^3^, ^13^, ^14^ In this study, we firstly explored the dynamic prognosis of metastatic clear cell renal cell carcinoma (mccRCC) patients who experienced cytoreductive surgery of primary tumor based on CS estimates.

## 2 MATERIALS AND METHODS

### 2.1 Data source and study population

Metastatic clear cell renal cell carcinoma cases were identified and extracted from the Surveillance, Epidemiology, and End Results (SEER) database through the SEER*Stat 8.3.9 software. We included the mccRCC patients treated with cytoreductive surgery of primary tumor between January 2004 and December 2015. The 10th revision of the International Classification of Disease for Oncology third edition (ICD‐O‐3) site codes (kidney: C64.9) and the histological type ICD‐O‐3 codes (clear cell adenocarcinoma type: 8310) were used for determining the mccRCC diagnosis. Tumor stage information of patients was collected and recorded in accord with the 6th edition of the American Joint Committee on Cancer Staging Manual. We included mccRCC patients aged ≥18 years treated with cytoreductive surgery of primary tumor. Exclusion criteria were as follows: patients diagnosed on autopsy; patients with incomplete follow‐up information.

### 2.2 Variables of interest and primary outcomes

General clinicopathological factors such as age at diagnosis, gender, ethnicity, marital status, type of surgery, histological subtype, pathological tumor stage (pT), pathological nodal stage (pN), Fuhrman nuclear grade, and follow‐up data like survival status were extracted. The primary outcomes of interest were 36‐month conditional overall survival (COS) and 36‐month conditional cancer‐specific survival (CCSS). Overall survival (OS) was defined as the interval from surgery to death of any causes and cancer‐specific survival (CSS) represented the time from surgery to death of mccRCC. The interval of OS or CSS ceased at the last time of follow‐up if patients were still alive or died of other causes.

### 2.3 Statistical analyses

CS probability was calculated by the formula, CS*_y_* = *S_x _*_+_ *_y_*/*S_x_*.^15^ Specifically, CS*_y_* indicates the additional *y* years survival probability in patients who had already survived *x* years. *S_x_* stands for the survival rate of *x* years quantified by Kaplan–Meier survival analysis. We applied Kaplan–Meier survival analysis to calculate COS and CCSS in all included mccRCC patients. Considering that age, pT stage, pN stage, and Fuhrman nuclear grade are the well‐established prognostic factors, subgroup CS stratified by the levels of above‐mentioned factors were performed.

To explore how the influence of aging and advanced tumor grade (pT3/4, pN1/2 and G3/4) changed with time, we performed a multivariate Cox proportional hazards regression in a subgroup stratified by the above established prognostic factors from time of diagnosis to 60 months after surgery. Hazard ratios (HRs) with 95% confidence intervals (CIs) for COS and CCSS were calculated. All statistical analyses were realized by SPSS (version 23.0; IBM) and GraphPad Prism (version 8.4.3; GraphPad Software). Two‐side with *p* value <0.05 was considered statistical significance.

## 3 RESULTS

### 3.1 General baseline demographics of the patients

From January 2004 to December 2015 in the SEER database, 4334 mccRCC patients once treated with cytoreductive surgery of primary tumor were identified. The median age of patients was 61 (IQR 52–68) years. The male and white population accounted for the majority of patients with 69.7% and 87.3%, respectively. Most of the patients (82.8%) experienced radical nephrectomy. With regard to pT and pN, most patients harbored pT3 (61.7%) and pN0 (73.4%). For Fuhrman nuclear grade, the percent of G1, G2, G3, and G4 was 1.8%, 19.7%, 40.4%, and 27.5%, respectively. More detailed information was displayed in Table 1.

**TABLE 1:** Baseline characteristics of included patients with metastatic clear cell renal cell carcinoma

Total patients, *n*	4334
Age at diagnosis, *n* (year)	
Median (IQR)	61 (52–68)
<65 year, *n* (%)	2720 (62.8)
≥65 year, *n* (%)	1614 (37.2)
Year of diagnosis, *n* (%)	
2004–2007	1227 (28.3)
2008–2011	1456 (33.6)
2012–2015	1651 (38.1)
Gender, *n* (%)	
Male	3020 (69.7)
Female	1314 (30.3)
Ethnicity, *n* (%)	
White	3782 (87.3)
Black	224 (5.2)
Other^a^	328 (7.5)
Marital status, *n* (%)	
Married	2897 (66.8)
Single	560 (12.9)
Divorced/separated	435 (10.0)
Widowed	301 (6.9)
Unknown	141 (3.4)
Type of surgery, *n* (%)	
Radical nephrectomy	3589 (82.8)
Complete/total/simple nephrectomy	524 (12.1)
Other^b^	221 (5.1)
pT, *n* (%)	
pT1	659 (15.2)
pT2	649 (15.0)
pT3	2675 (61.7)
pT4	292 (6.7)
Unknown	60 (1.4)
pN, *n* (%)	
pN0	3179 (73.4)
pN1/2	888 (20.5)
Unknown	267 (6.1)
Fuhrman nuclear grade, *n* (%)	
1	80 (1.8)
2	855 (19.7)
3	1753 (40.4)
4	1193 (27.5)
Unknown	453 (10.6)

Note

Other^a^ ethnicities included American Indian/Alaska native, Asian/Pacific Islander and other unspecified. Other^b^ types of surgery included excisional excision, local tumor destruction, cryosurgery, electrocautery, laser ablation, and photodynamic therapy.

### 3.2 COS in mccRCC

**FIGURE 1:**
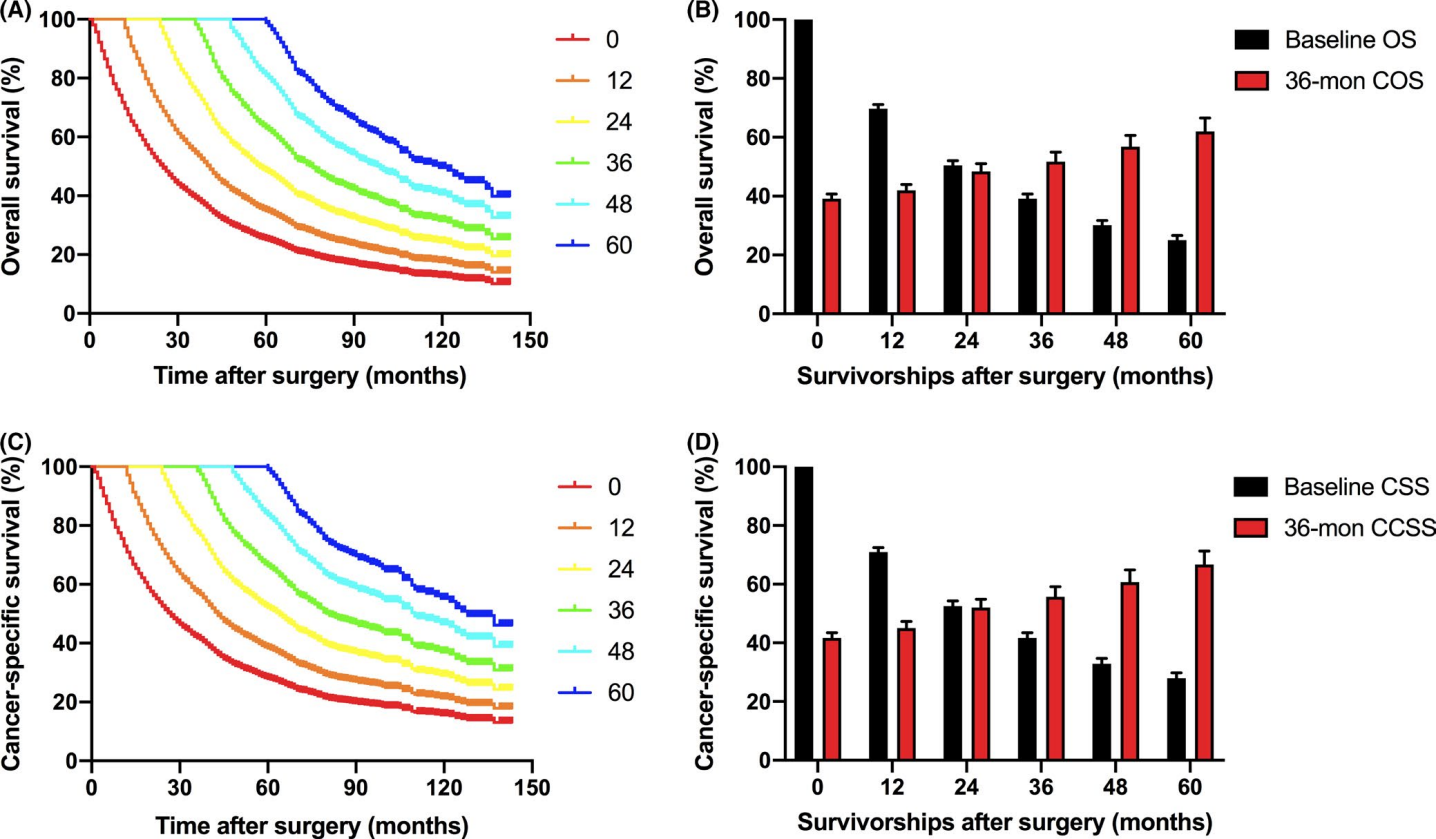
Conditional survival curves and the dynamic 36‐month COS/CCSS estimates relative to baseline OS/CSS estimates over time for metastatic clear cell renal cell carcinoma treated with cytoreductive surgery of primary tumor. (A) COS curve; (B) The dynamic 36‐month COS estimates relative to baseline OS estimates according to the time of survivorships; (C) CCSS curve; (D) The dynamic 36‐month CCSS estimates relative to baseline CSS estimates according to the time of survivorships. CCSS, conditional cancer‐specific survival; COS, conditional overall survival; CSS, cancer‐specific survival; OS, overall survival

The overall mortality of mccRCC patients during the follow‐up was 66.8% (2894), in which 54.0% (2339) of the patients experienced cancer‐specific mortality. The baseline OS estimations before surgery decreased from 69.7% (95% CI 68.2–71.1) to 25.1% (95% CI 23.5–26.6) at 12 and 60 months after surgery. However, the COS increased as the survivorship prolonged (Figure 1A; Table 2). The 12‐, 24‐, 36‐, 48‐, and 60‐month COS with an additional 12, 24, 36, 48, and 60 months of survivorship after surgery showed a gradually increasing trend. Particularly, the 36‐month COS improved by 3.3%–6.4% given per 12 additional months of survivorship, with 42.0%, 48.4%, 51.7%, 56.8%, and 62.0% for patients survived additional 12, 24, 36, 48, and 60 months after surgery (Figure 1B; Table 2).

**TABLE 2:** COS by time since cytoreductive surgery of primary tumor

Time (month)	No. at risk	COS %, (95% CI)				
		12	24	36	48	60
Baseline	4334	69.7 (68.2–71.1)	50.5 (48.9–52.1)	39.1 (37.5–40.7)	30.1 (28.5–31.7)	25.1 (23.5–26.6)
Time after surgery (month)						
12	2753	70.4 (68.6–72.1)	54.5 (52.5–56.4)	42.0 (39.9–44.0)	34.9 (32.8–37.0)	28.7 (26.6–30.8)
24	1760	75.6 (73.4–77.6)	58.2 (55.6–60.6)	48.4 (45.8–51.0)	39.8 (37.0–42.5)	34.0 (31.2–36.8)
36	1177	75.7 (73.0–78.1)	63.0 (59.9–65.9)	51.7 (48.4–54.9)	44.2 (40.8–47.6)	39.5 (35.9–43.0)
48	781	80.9 (77.8–83.6)	66.4 (62.7–69.9)	56.8 (52.7–60.6)	50.7 (46.4–54.8)	44.9 (40.3–49.4)
60	543	81.2 (77.5–84.4)	69.4 (65.0–73.4)	62.0 (57.1–66.5)	54.9 (49.5–60.0)	49.6 (43.7–55.2)

Abbreviation: COS, conditional overall survival.

**FIGURE 2:**
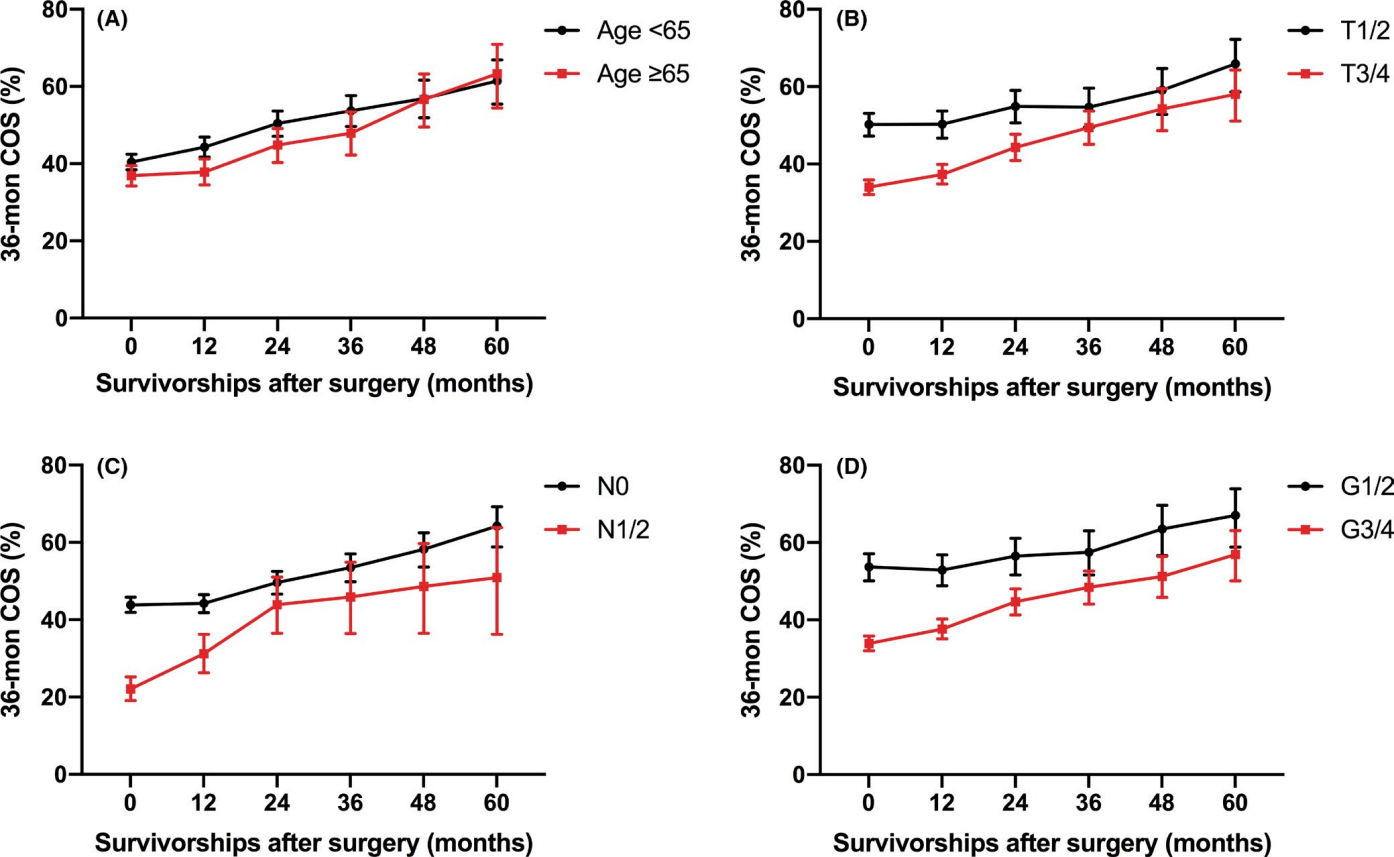
Dynamic 36‐month COS estimates according to the time of survivorships after surgery in metastatic clear cell renal cell carcinoma patients stratified by well‐established prognostic factors, (A) age, (B) pT stage, (C) pN stage, and (D) Fuhrman nuclear grade. COS, conditional overall survival

Given that age, pT stage, pN stage, and Fuhrman nuclear grade are the well‐established prognostic factors, 36‐month COS estimates were calculated in subgroups stratified by the levels of those prognostic factors. Figure 2 and Table 3 show an overall improvement in all subgroups as survival time increased. Patients aged <65 had better 36‐month COS than older patients who survived an additional 12, 24, and 36 months after surgery. However, the 36‐month COS of both age groups became similar at 48 and 60 months after surgery. The 36‐month COS of patients with pT1/2 improved from 50.2% (95% CI 47.2–53.1) at baseline to 65.9% (95% CI 58.6–72.2) at 60 months, which was better than those with pT3/4. Patients with pN1/2 suffered the worst 36‐month COS which ranged from 22.1% to 50.9%, while achieved the most significant survival gain compared to baseline, from 9.1% at 12 months to 28.8% at 60 months. Similarly, patients with advanced Fuhrman nuclear grade suffered worse 36‐month COS but harvested better survival gain compared to baseline.

**TABLE 3:** 36‐month COS estimates and survival gain after cytoreductive surgery of primary tumor

Cohort	Months survived					
	Baseline (0)	12	24	36	48	60
<65 year						
At risk, *n*	2720	1746	1132	778	521	368
36‐month COS, %	40.4	44.3	50.4	53.7	56.9	61.4
95% CI	38.4–42.4	41.7–46.9	47.1–53.6	49.6–57.6	51.9–61.6	55.4–66.9
Survival gain, %	—	3.9	10	13.3	16.5	21
≥65 year						
At risk, *n*	1614	1006	628	399	260	175
36‐month COS, %	36.9	37.8	44.8	47.9	56.6	63.3
95% CI	34.2–39.5	34.5–41.2	40.3–49.1	42.2–53.4	49.5–63.2	54.4–70.9
Survival gain, %	—	0.9	7.9	11	19.7	26.4
pT1/2						
At risk, *n*	1307	956	671	478	338	242
36‐month COS, %	50.2	50.3	54.9	54.7	59.1	65.9
95% CI	47.2–53.1	46.7–53.7	50.6–59.0	49.5–59.6	52.8–64.7	58.6–72.2
Survival gain, %	—	0.1	4.7	4.5	8.9	15.7
pT3/4						
At risk, *n*	2967	1751	1058	680	432	292
36‐month COS, %	34	37.3	44.3	49.4	54.2	58
95% CI	32.1–35.9	34.8–39.9	40.9–47.7	45.1–53.7	48.6–59.5	51.1–64.3
Survival gain, %	—	3.3	10.3	15.4	20.2	24
pN0						
At risk, *n*	3179	2166	1437	968	640	439
36‐month COS, %	43.8	44.2	49.6	53.5	58.2	64.2
95% CI	41.9–45.8	41.8–46.5	46.6–52.5	49.8–57.0	53.6–62.5	58.8–69.2
Survival gain, %	—	0.4	5.8	9.7	14.4	20.4
pN1/2						
At risk, *n*	888	422	213	132	90	67
36‐month COS, %	22.1	31.2	43.9	45.9	48.6	50.9
95% CI	19.1–25.2	26.3–36.2	36.5–51.0	36.4–54.9	36.5–59.7	36.2–63.8
Survival gain, %	—	9.1	21.8	23.8	26.5	28.8
Grade 1/2						
At risk, *n*	935	719	514	369	259	195
36‐month COS, %	53.7	52.9	56.5	57.5	63.5	67
95% CI	50.1–57.1	48.8–56.8	51.6–61.1	51.6–63.0	56.6–69.6	58.8–73.9
Survival gain, %	—	−0.8	2.8	3.8	9.8	13.3
Grade 3/4						
At risk, *n*	2946	1733	1054	688	452	304
36‐month COS, %	33.9	37.6	44.7	48.4	51.2	56.9
95% CI	32.0–35.8	35.1–40.2	41.3–48.0	44.1–52.6	45.8–56.4	50.1–63.1
Survival gain, %	—	3.7	10.8	14.5	17.3	23

Abbreviation: COS, conditional overall survival.

### 3.3 CCSS in mccRCC

Similar to the COS analyses, the baseline CSS estimations decreased while the CCSS increased with time (Figure 1C; Table 4). The 12‐, 24‐, 36‐, 48‐, and 60‐month CCSS at 12, 24 36, 48, and 60 months were all higher than the baseline CSS estimations. Specifically, the 36‐month CCSS increased from 41.7% (95% CI 39.9–43.4) estimated at baseline to 67.1% (95% CI 62.0–71.7) in patients who had already survived 60 months (Figure 1D; Table 4).

**TABLE 4:** CCSS by time since cytoreductive surgery of primary tumor

Time (month)	No. at risk	CCSS %, (95% CI)				
		12	24	36	48	60
Baseline	3788	71.0 (69.4–72.5)	52.6 (50.8–54.3)	41.7 (39.9–43.4)	32.9 (31.1–34.7)	28.0 (26.2–29.8)
Time after surgery (month)						
12	2399	72.0 (70.0–73.8)	57.1 (54.9–59.2)	45.1 (42.8–47.3)	38.3 (36.0–40.6)	32.3 (29.9–34.6)
24	1530	77.5 (75.2–79.6)	61.2 (58.5–63.8)	52.1 (49.2–54.9)	43.8 (40.8–46.8)	37.9 (34.8–41.0)
36	1032	77.8 (75.0–80.3)	66.2 (62.9–69.3)	55.7 (52.1–59.2)	48.2 (44.4–51.9)	44.9 (41.0–48.7)
48	682	83.4 (80.3–86.1)	70.2 (66.3–73.9)	60.7 (56.3–64.8)	56.6 (52.0–61.0)	52.0 (47.0–56.8)
60	475	83.3 (79.4–86.5)	72.0 (67.3–76.2)	67.1 (62.0–71.7)	61.6 (56.0–66.8)	55.2 (48.7–61.3)

Abbreviation: CCSS, conditional cancer‐specific survival.

**FIGURE 3:**
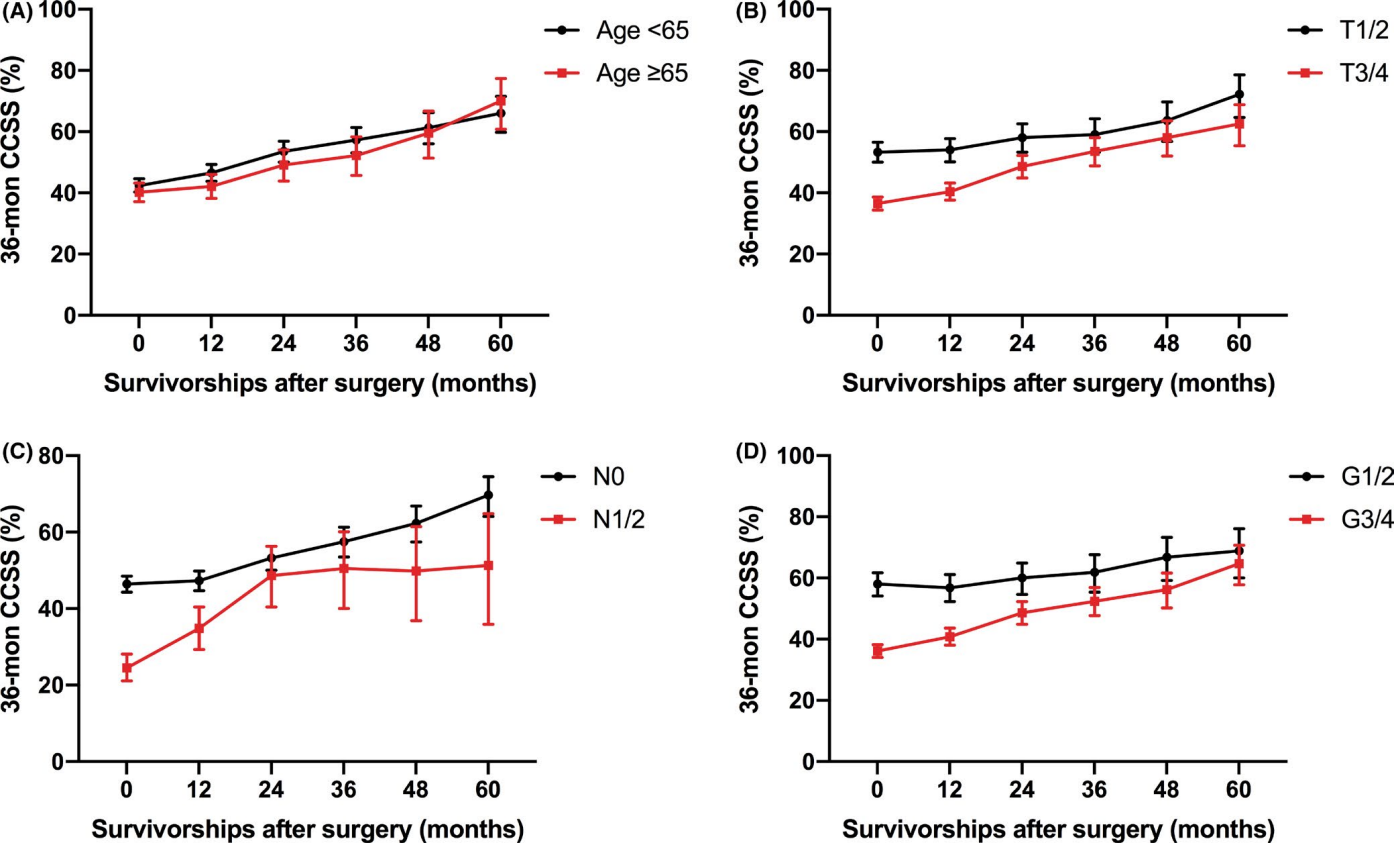
Dynamic 36‐month CCSS estimates according to the time of survivorships after surgery in metastatic clear cell renal cell carcinoma patients stratified by well‐established prognostic factors, (A) age, (B) pT stage, (C) pN stage, and (D) Fuhrman nuclear grade. CCSS, conditional cancer‐specific survival

We also performed subgroup analyses based on age, pT stage, pN stage, and Fuhrman nuclear grade. Patients aged <65 had better 36‐month CCSS than patients aged ≥65, while the difference between the two groups reduced gradually over time. As for tumor stage, although the 36‐month CCSS of patients with more advanced tumor stage (pT3/4, pN1/2, and G3/4) was worse, the better survival gain was harvested. Figure 3 and Table 5 show the more detailed information.

**TABLE 5:** 36‐month CCSS estimates and survival gain after cytoreductive surgery of primary tumor

Cohort	Months survived					
	Baseline (0)	12	24	36	48	60
<65 year						
At risk, *n*	2476	1583	1025	707	470	332
36‐month CCSS, %	42.4	46.5	53.5	57.3	61.3	66
95% CI	40.3–44.6	43.8–49.3	50.0–56.9	53.0–61.4	56.0–66.2	59.8–71.5
Survival gain, %	—	4.1	11.1	14.9	18.9	23.6
≥65 year						
At risk, *n*	1312	816	505	325	212	143
36‐month CCSS, %	40.2	42.1	49.1	52.2	59.5	70
95% CI	37.1–43.2	38.2–46.0	43.9–54.0	45.7–58.3	51.4–66.7	60.8–77.4
Survival gain, %	—	1.9	8.9	12	19.3	29.8
pT1/2						
At risk, *n*	1116	811	569	410	286	202
36‐month CCSS, %	53.3	54.0	58.0	59.0	63.6	72.2
95% CI	50.0–56.5	50.1–57.7	53.3–62.5	53.3–64.2	56.8–69.7	64.6–78.5
Survival gain, %	—	0.7	4.7	5.7	10.3	18.9
pT3/4						
At risk, *n*	2628	1554	938	608	389	267
36‐month CCSS, %	36.5	40.4	48.6	53.5	58	62.5
95% CI	34.4–38.6	37.6–43.2	44.9–52.2	48.8–58.0	52.0–63.5	55.4–68.8
Survival gain, %	—	3.9	12.1	17	21.5	26
pN0						
At risk, *n*	2770	1886	1247	850	560	383
36‐month CCSS, %	46.4	47.3	53.2	57.5	62.3	69.7
95% CI	44.3–48.5	44.7–49.8	50.0–56.3	53.5–61.3	57.4–66.8	64.1–74.5
Survival gain, %	—	0.9	6.8	11.1	15.9	23.3
pN1/2						
At risk, *n*	791	373	186	115	81	60
36‐month CCSS, %	24.5	34.8	48.6	50.5	49.8	51.3
95% CI	21.1–28.1	29.3–40.4	40.4–56.3	40.0–60.1	36.8–61.4	35.9–64.8
Survival gain, %	—	10.3	24.1	26.0	25.3	26.8
Grade 1/2						
At risk, *n*	801	615	449	324	224	166
36‐month CCSS, %	58.0	56.8	60.0	61.9	66.8	68.9
95% CI	54.1–61.7	52.3–61.1	54.6–64.9	55.4–67.6	59.2–73.3	60.0–76.1
Survival gain, %	—	−1.2	2.0	3.9	8.8	10.9
Grade 3/4						
At risk, *n*	2597	1529	921	602	398	270
36‐month CCSS, %	36.1	40.8	48.6	52.4	56.2	64.7
95% CI	34.0–38.2	38.0–43.6	44.9–52.3	47.7–56.9	50.2–61.6	57.8–70.7
Survival gain, %	—	4.7	12.5	16.3	20.1	28.6

Abbreviation: CCSS, conditional cancer‐specific survival.

### 3.4 Multivariable Cox regression analyses

To determine the evolution of traditional prognostic factors on COS, multivariable Cox regression analysis was applied (Table S1). Age, pT stage, pN stage, and Fuhrman nuclear grade were all identified as independent OS predictors at baseline, with HR 1.14 (95% CI 1.05–1.24), 1.31 (95% CI 1.19–1.44), 1.72 (95% CI 1.56–1.89), and 1.53 (95% CI 1.38–1.69), respectively. However, none of them was considered as a continuously predictor on COS over time. Age ≥65 years was a risk factor for overall mortality until 36 months additional survivorship after surgery. A similar trend of adverse pathological features (pT3/4 and pN1/2) were also observed. The influence of advanced Fuhrman nuclear grade (G3/4) remained significant until 60 months after surgery, with HR 1.43 (95% CI 1.26–1.62) at 12 months, 1.35 (95% CI 1.16–1.59) at 24 months, 1.32 (95% CI 1.08–1.62) at 36 months, 1.31 (95% CI 1.01–1.71) at 48 months, and 1.32 (95% CI 0.94–1.85) at 60 months, respectively.

A multivariable Cox regression model was also conducted to investigate the effect of prognostic variables on CCSS (Table S2). All of the four factors except advanced age were independent predictors for CSS at baseline. However, neither tumor stage (pT) nor Fuhrman nuclear grade was a continuously prognostic factor on CCSS. Only pN1/2 was consistently correlated with worse CCSS during the follow‐up.

## 4 DISCUSSION

In general, survival prediction at diagnosis is usually employed to guide the selection of treatment and making surveillance strategy. However, limited information could be provided to predict the chance of additional survival period in patients who outlive the predicted time. For example, if a cancer patient who has already survived 5 years after treatment wants to know how the disease will impact his/her survival in the future years, the clinicians cannot counsel the patient with a detail information due to less clinical data. In such a context, CS estimates based on the concept of conditional probability is introduced.^15^ Given that the survival time patients have already achieved, CS is commonly applied to estimate the survival of cancer patients. CS provides more personalized prediction of prognosis which might be valuable in counseling patients and making surveillance strategies.

In 2003, Skuladottir and colleagues pioneered the concept of CS in lung cancer patients.^16^ They found that the additional 5‐year survival probability improved from 33%–36% at first year to 60%–67% at fifth year after treatment in all patients with lung cancer, which was much more favorable than 5‐year survival (<10%) estimated at diagnosis without considering the changes of survival rate as the time of survivorship increased. Then, CS aroused the attention of researchers who focused on prognostic studies in cancers. Swords et al reported that cancer‐specific mortality of patients with pancreaticductal adenocarcinoma peaked at 3 years after diagnosis, and then decreased in the following years until at 13 years after diagnosis and remained stable at <3%.^8^ Similar results were also found in other malignancies.^10^, ^17^, ^18^, ^19^, ^20^ As for mRCC, which is an advanced stage of the disease with poor prognosis, the CS studies are limited. And major of them assessed the CS of mRCC in a systemic or targeted therapy setting.^3^, ^13^, ^21^ As one of the recommended treatments for mRCC patients with good performance status evidenced by several studies,^14^, ^22^, ^23^, ^24^, ^25^ no data on CS of mccRCC who underwent cytoreductive surgery of primary tumor have been reported before. For this unmet need, we performed the study.

In our study, increasingly improvement of COS and CCSS was observed in all mccRCC patients as survival time increased. Of note, the 36‐month COS and 36‐month CCSS improved from 42.0% and 45.1% at 12 months to 62.0% and 67.1% at 60 months after surgery. Compared to estimations at diagnosis, +22.9% and +25.4% survival gain of 36‐month COS and 36‐month CCSS were harvested at 60 months after surgery. For well‐known prognostic factors, age, pT stage, pN stage, and Fuhrman nuclear grade, the prognostic value of them all converged over time.

Xing et al found 5‐year CS estimate of stage I melanoma remained stable at ~97% with time. However, patients with more advanced stage melanoma (stages II, III, and IV) at 5 years achieved significant survival gain in 5‐year CS estimates, with +14%, +36% and +65% compared to baseline, respectively.^20^ In one study focused on patients treated with radical prostatectomy, researchers reported that the 5‐year conditional disease‐free survival rate of patients with pT3b‐4 improved from 20.7% at baseline to 78.9% at 4 years of survivorship. While only +12.5% survival gain was observed in patients with pT2.^11^ In patients with non‐metastatic RCC, a study from Canada illustrated that patients with pT4N0/pTanyN1 benefited most in 5‐year CCSS from 46.8% at baseline to 77.9% at 8 years survived, whereas the 5‐year CCSS of pT1aN0 patients changed little with time.^26^ Similar results were recorded in another study.^27^ With regard to mRCC, Harshman et al revealed that 2‐year COS of patients in poor‐risk group climbed continuously from 11% to 33% during the 18 years of follow‐up, while the changes of patients in favorable and intermediate groups were not significant.^13^ Kang and his team also reported that, compared to patients at favorable or intermediate risk, mRCC patients at poor risk benefited more in 36‐month COS at all survival periods since the beginning of targeted therapy.^3^ In our study, similar results were observed. That was, much more survival gain of 36‐month COS and CCSS at all follow‐up points were achieved in patients with aggressive disease (age >65, pT3/4, pN1‐2, and G3/4) compared to their counterparts. In addition, it was exciting to find that the gaps of CS in each subgroup stratified by age, pT, pN, and Fuhrman nuclear grade became narrowing as the survival time increased, which was helpful to rebuild the positive attitude toward overcoming the disease in patients with mccRCC. Also, the findings might be valuable for physicians to make more individualized surveillance strategies.

Prognostic factors are the key issues that physicians pay attention to when counseling patients with prognosis. To explore the changeable significance of four well‐known prognostic factors (age, pT stage, pN stage, and Fuhrman nuclear grade) on COS and CCSS, multivariable Cox regression analyses were applied at baseline, 12, 24, 36, 48, and 60 months after surgery. Our results indicated that the impact of prognostic factors changed dynamically as the duration of survivorship increased. Although the four factors were all associated with OS at baseline, the significance of them decreased with time and disappeared finally. Similar results were obtained in CCSS. The decreasing trend was also evidenced by other studies.^3^, ^7^, ^18^, ^26^ The similar findings above imply that personalized surveillance strategies should be made depending on different disease statuses. For example, although we found the CCSS of mccRCC patients with pN1/2 was continuously improved and the difference between the two subgroups (pN0 vs. pN1/2) became narrowing in our study, nodal invasion was still a risk factor of cancer‐specific mortality at 60 months. In other words, more rigorous surveillance scheme was required at least in the following 5 years after surgery. While for patients with pT3/4, an intensive surveillance in the first 2 years after surgery was suggested.

To the best of our knowledge, our work is the first CS study on mccRCC treated with cytoreductive surgery of a primary tumor. Our findings firstly uncover the dynamic survival profiles in mccRCC patients treated with cytoreductive surgery as survival time progresses. In our study, we found the CS of all patients improved with survival period increasing regardless of disease status. To provide more individualized information for prognosis estimating and surveillance scheme making, 36‐month CS estimations were calculated in mccRCC patients stratified by age, pT stage, pN stage, and Fuhrman nuclear grade. Furthermore, we also revealed the evolution of prognostic factors with time. It is believed that our data could provide valuable and practical information for patients counseling and surveillance strategies making.

However, several limitations in our study should be acknowledged. First, as a retrospective cohort study based on the SEER database, high potential of selection biases should be considered. Second, detailed data on systematic therapy including targeted therapy and immunotherapy are not available in SEER database, which impedes further subgroup analyses. Third, due to the limited records, we could not perform subgroup analyses according to the sites or number of metastases and the status of comorbidities or physical performance. Fourth, because of the major population in SEER database is North American, our results should be consulted to other population with cautiousness.

## 5 CONCLUSION

In summary, COS and CCSS improved with time dynamically in all mccRCC patients treated with cytoreductive surgery of primary tumor. Patients with advanced disease achieved significant survival gain and even could harvest a better prognosis given that the survivorships exceed a certain period. The significance of prognostic factors could diminish and even disappear in long‐term survivorship. Our findings could provide valuable and practical data for patients counselling and surveillance strategy making.

## CONFLICT OF INTEREST

All authors certify that they have no conflict of interest to declare.

## ETHICAL APPROVAL STATEMENT

All data analyzed in this study were publicly available, from the Surveillance, Epidemiology, and End Results (SEER) database (https://seer.cancer.gov/). No ethical approval and patient consent are required.

## Supporting information

Table S1‐S2

## Data Availability

The data analyzed in this study were publicly available, from the Surveillance, Epidemiology, and End Results (SEER) database (https://seer.cancer.gov/).
