# Supplementary material for: Conditional survival of metastatic clear cell renal cell carcinoma: How prognosis evolves after cytoreductive surgery of primary tumor
Source: Cancer Med. 2021 Sep 12;10(21):7492–502. doi: 10.1002/cam4.4270 (PMC8559515; doi:10.1002/cam4.4270)
Supplement: Supplementary file 1 — Table S1‐S2 [file CAM4-10-7492-s001.docx]

Supplementary table 1. Multivariable Cox proportional hazards regression predicting overall mortality in mccRCC patients treated with cytoreductive surgery of primary tumor.

|  | Months Survived | | | | | |
| --- | --- | --- | --- | --- | --- | --- |
|  | Baseline (0) | 12 | 24 | 36 | 48 | 60 |
| Age ≥65 yr (Ref: ＜65 yr) | | | | | | |
| HR (95% CI) | 1.14 (1.05-1.24) | 1.20 (1.07-1.34) | 1.18 (1.02-1.37) | 1.14 (0.94-1.39) | 1.18 (0.91-1.53) | 1.22 (0.87-1.70) |
| *p*-value | 0.003 | 0.002 | 0.032 | 0.192 | 0.219 | 0.245 |
| pT3/4 (Ref: pT1/2) | | | | | | |
| HR (95% CI) | 1.31 (1.19-1.44) | 1.20 (1.06-1.34) | 1.15 (0.99-1.34) | 1.11 (0.91-1.35) | 1.04 (0.81-1.34) | 0.99 (0.72-1.38) |
| *p*-value | <0.001 | 0.003 | 0.067 | 0.302 | 0.761 | 0.971 |
| pN1/2 (Ref: pN0) | | | | | | |
| HR (95% CI) | 1.72 (1.56-1.89) | 1.45 (1.27-1.66) | 1.26 (1.04-1.53) | 1.20 (0.93-1.54) | 1.28 (0.92-1.77) | 1.53 (1.02-2.28) |
| *p*-value | <0.001 | <0.001 | 0.018 | 0.167 | 0.143 | 0.04 |
| Grade 3/4 (Ref: Grade 1/2) | | | | | | |
| HR (95% CI) | 1.53 (1.38-1.69) | 1.43 (1.26-1.62) | 1.35 (1.16-1.59) | 1.32 (1.08-1.62) | 1.31 (1.01-1.71) | 1.32 (0.94-1.85) |
| *p*-value | <0.001 | <0.001 | <0.001 | 0.007 | 0.04 | 0.106 |

Supplementary table 2. Multivariable Cox proportional hazards regression predicting cancer-specific mortality in mccRCC patients treated with cytoreductive surgery of primary tumor.

|  | Months Survived | | | | | |
| --- | --- | --- | --- | --- | --- | --- |
|  | Baseline (0) | 12 | 24 | 36 | 48 | 60 |
| Age ≥65 yr (Ref: ＜65 yr) | | | | | | |
| HR (95% CI) | 1.09 (0.99-1.21) | 1.13 (1.00-1.29) | 1.13 (0.95-1.35) | 1.06 (0.84-1.33) | 1.10 (0.81-1.49) | 1.11 (0.75-1.63) |
| *p*-value | 0.072 | 0.055 | 0.153 | 0.623 | 0.536 | 0.599 |
| pT3/4 (Ref: pT1/2) | | | | | | |
| HR (95% CI) | 1.32 (1.19-1.47) | 1.23 (1.08-1.41) | 1.20 (1.01-1.42) | 1.11 (0.89-1.39) | 1.07 (0.80-1.43) | 1.19 (0.81-1.74) |
| *p*-value | <0.001 | 0.002 | 0.044 | 0.342 | 0.656 | 0.376 |
| pN1/2 (Ref: pN0) | | | | | | |
| HR (95% CI) | 1.75 (1.58-1.94) | 1.45 (1.25-1.69) | 1.31 (1.06-1.62) | 1.26 (1.08-1.67) | 1.48 (1.04-2.11) | 1.73 (1.12-2.68) |
| *p*-value | <0.001 | <0.001 | 0.012 | 0.011 | 0.029 | 0.013 |
| Grade 3/4 (Ref: Grade 1/2) | | | | | | |
| HR (95% CI) | 1.61 (1.43-1.81) | 1.52 (1.31-1.75) | 1.37 (1.14-1.64) | 1.29 (1.03-1.63) | 1.29 (0.96-1.75) | 1.24 (0.85-1.82) |
| *p*-value | <0.001 | <0.001 | 0.001 | 0.028 | 0.092 | 0.268 |
